# Exploring influenza vaccination coverage and barriers to influenza vaccine uptake among preschool children in Fuzhou in 2022: a cross-sectional study

**DOI:** 10.3389/fpubh.2025.1588760

**Published:** 2025-07-02

**Authors:** Haimei Jia, Wenyan Gao, Xun Huang, Qinghua Wang, Yonghan Huang, Liang Chen, Desi Zheng, Yinchuan Zhang, Lifei Xu

**Affiliations:** ^1^Fuzhou Center for Disease Control and Prevention, Affiliated with Fujian Medical University, Fuzhou, China; ^2^Gulou District Center for Disease Control and Prevention, Fuzhou, China; ^3^Fuqing Center for Disease Control and Prevention, Fuzhou, China; ^4^Minqing County Center for Disease Control and Prevention, Fuzhou, China; ^5^Yongtai County Center for Disease Control and Prevention, Fuzhou, China

**Keywords:** influenza, preschool children, parental attitudes, influenza vaccination coverage, questionnaire survey

## Abstract

**Background:**

Children are vulnerable to influenza virus due to their developing immune systems, particularly children aged 6 months-5 years (preschool children). To improve the uptake of influenza vaccine in preschool children, it is important to determine the influencing factors of Chinese parents/guardians’ (P/Gs) intention and behavior for children to receive. We implemented an investigation to determine coverage of influenza vaccination in preschool children and the influencing factors of being vaccinated against influenza among preschool children in Fuzhou.

**Methods:**

This is a cross-sectional study. Using a hierarchical approach, based on the coverage of influenza vaccination in preschool children, the 12 districts/counties in Fuzhou were divided into two levels. In each level, two urban districts and two counties were selected, including 2 randomly selected vaccination clinics and 2 kindergartens. A standardized anonymous questionnaire was used to collect information on P/Gs. Chi-square testing and multivariate logistic regression were used to analyze factors that may be associated with influenza.

**Results:**

The coverage rate of influenza vaccination was 7.38% among preschool children in 2022 in Fuzhou City. A total of 8,768 guardians completed the questionnaire. 54.70% of the responders had received at least one dose of flu. Only 23.56% of the P/Gs involved were able to correctly list the influenza clinical feature. Higher education status had higher coverage (*p*-values < 0.05). Multivariate analysis showed birth order [odds ratio (OR) = 0.76, 95% confidence interval (CI): 0.63, 0.92], medical insurance [OR = 1.42, 95% CI: 1.22, 1.65], occupation [OR = 0.84, 95% CI: 0.75, 0.93], average monthly household income ≥ 10,000 [OR = 0.66, 95% CI: 0.56, 0.79], vaccine prices > 200 [OR = 1.66, 95% CI: 1.41, 1.97], and total duration of each vaccination session [OR = 0.49, 95% CI: 0.42, 0.58] were associated with flu vaccination.

**Conclusion:**

Influenza vaccination coverage among preschoolers was low, and parental/guardian knowledge regarding influenza prevention was inadequate. Enhanced awareness, vaccine understanding, and recommendation policies correlated with higher coverage. Authorities should implement sustainable financing and incentives to ensure access and affordability, while promoting education to convert vaccination intentions into actual uptake.

## Introduction

Influenza remains a major cause of morbidity, mortality, and economic burden worldwide each year. There are approximately 1 billion cases of influenza worldwide each year, of which 3 to 5 million are severe cases, resulting in 290,000–650,000 deaths globally (1). Children are relatively immunologically naive to influenza virus, leading to increased morbidity on infection (2). Among children under 5 years globally, there were an estimated one hundred million influenza virus episodes, and results in a substantial burden on health services worldwide (3). Lai et al. (4) indicated that the average economic burden of children due to influenza-like illness was 1,647 yuan (237.2 dollars) per episode, and the indirect economic burden due to the loss of caregivers’ labor time also was fairly large. The estimated overall attack rate in China was reported to be approximately 5.5% in all age groups, with the highest attack rate observed in 0–4 years old preschool children (31.9%). However, influenza vaccination coverage among children in China is low, remaining at approximately 25% (5).

World Health Organization (WHO) and European Union targets for immunization rates for at-risk populations are 75% (6). Both the Advisory Committee on Immunization Practices and Chinese Center for Disease Control and Prevention were simultaneously recommending universal influenza vaccination for preschool children (between the ages of 6 and 59 months), as well as those with high-risk conditions (7, 8). Vaccinating children against influenza could not only protect children themselves but also protect the whole community and reduce influenza incidence in the general population (9).

Parents/guardians (P/Gs) are the primary decision-makers for all family behaviors, it is critical to understand the factors that influence P/Gs’ intentions to vaccinate their children. Low MSF’s article showed that P/Gs’ willingness to vaccinate is a strong predictor of child influenza vaccination (10). Thus, a comprehensive survey of P/Gs is warranted to assess vaccination willingness and identify potential determinants influencing their decision-making.

In order to better control the influenza prevalent among preschool children, Fuzhou Health Commission and Fuzhou Center for Disease Control and Prevention provide health reminders and recommend getting vaccinated against influenza each year. However, the actual vaccination willingness of preschool children and the factors influencing their vaccination remain unclear. During 2022 through 2023, we conducted a field investigation to evaluate the knowledge, attitudes of P/Gs regarding this influenza vaccination, and assess their status of influenza vaccination. This study was designed to identify factors affecting influenza vaccination among preschool children and provide evidence increasing influenza vaccination rates among preschool children.

## Methods

### Study design and setting

Data on influenza vaccinations of children aged 6 months-5 years (preschool children) in 2022 were obtained from the Fujian Province Immunization Information System, which contains vaccination data for all citizens living in Fuzhou. Population data used in this study were obtained from the China Information System for Disease Control and Prevention.

### Setting and subjects

Fuzhou area is made of 12 urban districts (counties). Based on the coverage of influenza vaccination in preschool children, the 12 districts/counties in Fuzhou were divided into two levels A and B (A: high coverage rate > 8.0%; B: low coverage rate < 8.0%) In each level, two urban districts and two counties were selected, including 2 randomly selected vaccination clinics and 2 kindergartens. There was no specific influenza vaccination campaign during the investigation period. At least 2,700 P/Gs of preschool children were investigated in each level. P/Gs who were unwilling to participate in the study and children with contraindications of influenza vaccines were excluded.

### Data collection

A standardized anonymous questionnaire designed specifically for the study was used to collect information, including fundamental demographic details concerning the infants and their families (sex, age, household composition, education status of the P/Gs, health status of children, health insurance status, P/Gs occupation, average monthly household income, birth information of children), influenza vaccination status of children, health-related beliefs and attitudes to influenza vaccination knowledge to influenza and influenza vaccination, and the demand for vaccination services (trust and satisfaction with vaccination staff, schedule of clinics, medical service environment). The questionnaire was distributed by investigators, filled in, and retrieved immediately at the field.

### Data analysis

Knowledge of influenza and influenza vaccination: Assign 1 point to each question, 1 point for correct answers, and 0 points for errors, the total score is 14. The scores were categorized into three groups: 0–5 unknown, 6–10 general know, 11–14 good know, with a hierarchy describing the respondent’s state of knowledge. We established a database for analysis with SPSS version 26.0. Coverage of influenza vaccination, demographics, knowledge about influenza and influenza vaccination, and health-related beliefs and attitudes to influenza vaccination were analyzed descriptively. Chi-square testing and multivariate logistic regression were used to analyze factors that may be associated with influenza vaccination. We used a 2-tail *p*-value (*p*) significance level of 0.05.

## Results

### Vaccination rates for flu in Fuzhou

Data obtained from the Fujian province immunization information system showed the total coverage of influenza vaccination (at least one dose) among preschool children in 2022 was 7.38%. The coverage was between 4.38–10.76% among 12 urban districts (counties). The coverage of influenza vaccination in preschool children among A (8.77%) was higher than in level B (6.10%) (*p* < 0.05).

### Demographic characteristics

A total of 8,768 P/Gs were surveyed, their median age was 33 years (range: 20–99), and most participants were mothers (81.23%). According to occupational categorization, 45.02% of the respondents were enterprises and institutions, 59.39% were three-year college/university, and 61.54% had legal holidays.

7.80% (684) were 6–11 months children, 20.78% (1,822) were between 1–2 years, 71.42% (6,262) were 3–6 years, 52.16% (4,573) were males. 51.45% of these were from the second birth, 90.81% had health insurance. Demographics and other characteristics are illustrated in Table 1.

**Table 1:** Demographic characteristics of parents/guardians and children.

Variable	Levels	Number of respondents	Percent %
Parents/guardians			
Age group (years)	20-30	2,598	29.63
	31-40	5,290	60.33
	>41	880	10.04
Fill the questionnaire	Mother	7,123	81.23
	Father	1,385	15.80
	Grandparents	138	1.57
	Other	122	1.39
Occupation	Enterprises and public institutions	3,947	45.02
	Self-employed/farmer	1,599	18.24
	Unemployed/other	3,222	36.75
Education level	Middle school or below	1,273	14.52
	High school	2,261	25.79
	Three-year college/university	4,944	56.39
	Master and above	290	3.31
Average monthly household income (RMB)	<3,000	870	9.92
	3,000-4,999	2,344	26.73
	5,000-9,999	3,379	38.54
	≥10,000	2,175	24.81
Rest according to legal holidays	Yes	5,396	61.54
	Occasional overtime	2,389	27.25
	Frequent overtime	983	11.21
Transportation to the Vaccination Clinic	Walking	1,065	12.15
	Bicycle	104	1.19
	Motorcycle/electric vehicle	4,089	46.64
	Public transportation	301	3.43
	Self-driving	3,209	36.60
One-way inoculation time (minutes)	<30	7,540	85.99
	30-60	1,143	13.04
	>60	85	0.97
Children			
Age group	6-11 months	684	7.80
	1-2 years	1,822	20.78
	3-6 years	6,262	71.42
Gender	Male	4,573	52.16
	Female	4,195	47.84
Birth order	First	3680	41.97
	Second	4511	51.45
	Third or more	577	6.58
Common cold	Often	1220	13.91
	Infrequent	6011	68.56
	Seldom	1537	17.53
Medical insurance	No	806	9.19
	Purchased	7962	90.81

### Knowledge regarding influenza

**Figure 1:**
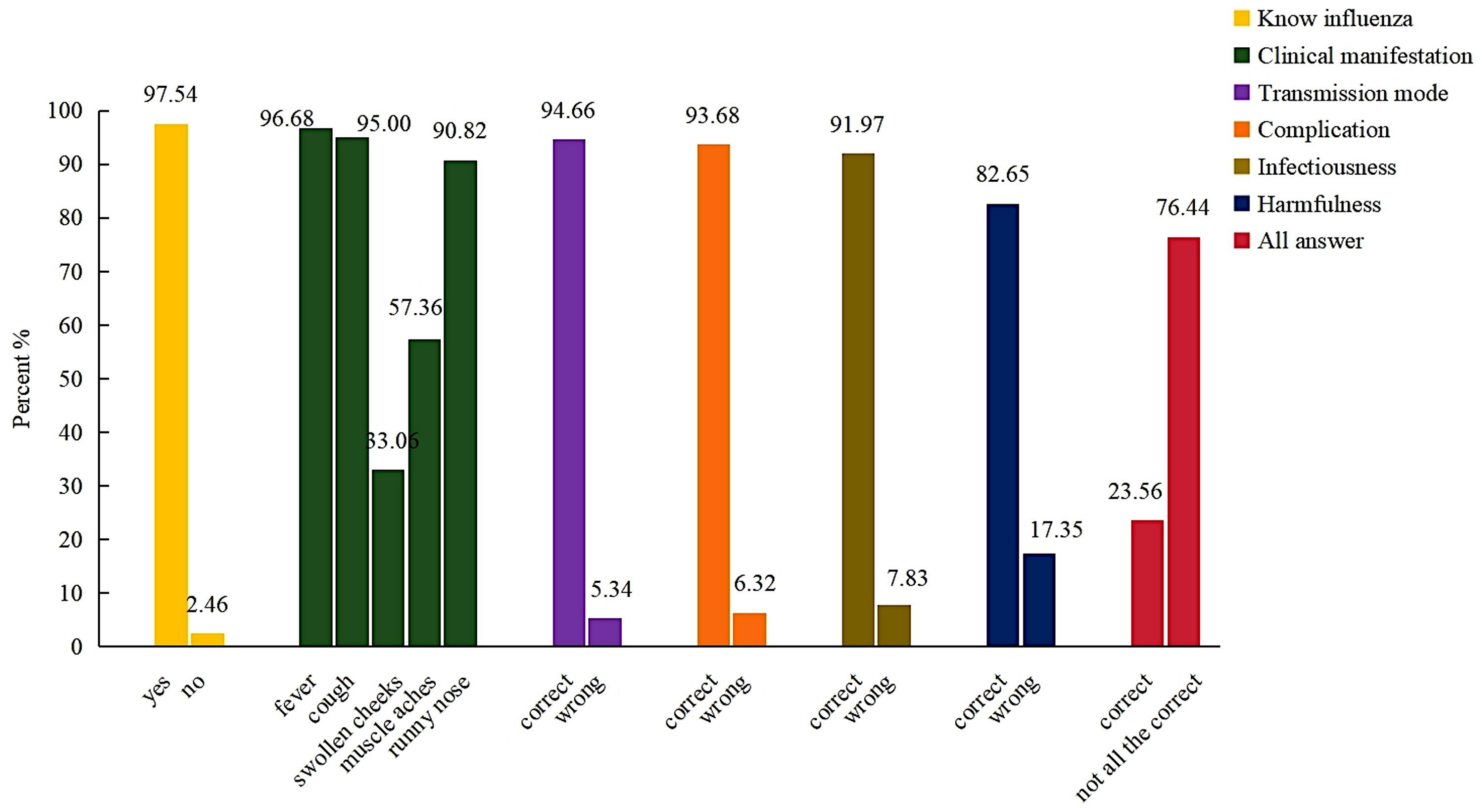
Respondents’ answers about influenza knowledge.

Almost all of the respondents (97.54%) answered they knew the flu, however, 23.56% of the P/Gs involved were able to correctly list the influenza clinical feature. And the percentage with correct knowledge of transmission mode, complication, and the infectiousness of influenza were 94.66, 93.68, and 91.97%, respectively (Figure 1).

31–40 years old P/Gs had better knowledge about influenza than younger P/Gs and older P/Gs (24.40, 21.86, and 23.52%, respectively, *p* < 0.05). When we compared the education status, there were differences among P/Gs, 28% of P/Gs with Three-year college/university and above had answered correctly, which was higher than those with a high school education (19.46%) and middle school or below (13.12%; *p* < 0.05) (Table 2).

**Table 2:** Main reasons influencing respondents’ knowledge of influenza.

Variable	Levels	The rate of correct answer (%)	*χ^2^*	*p*
Hierarchy	A	23.29	0.38	0.54
	B	23.86		
P/Gs age	20–30	21.86	6.21	0.04
	31–40	24.40		
	>41	23.52		
Education level	Middle school or below	13.12	152.75	0.00
	High school	19.46		
	Three-year college/university	27.97		
	Master’s degree and above	26.21		

### Influenza vaccination and vaccination rates for flu

**Figure 2:**
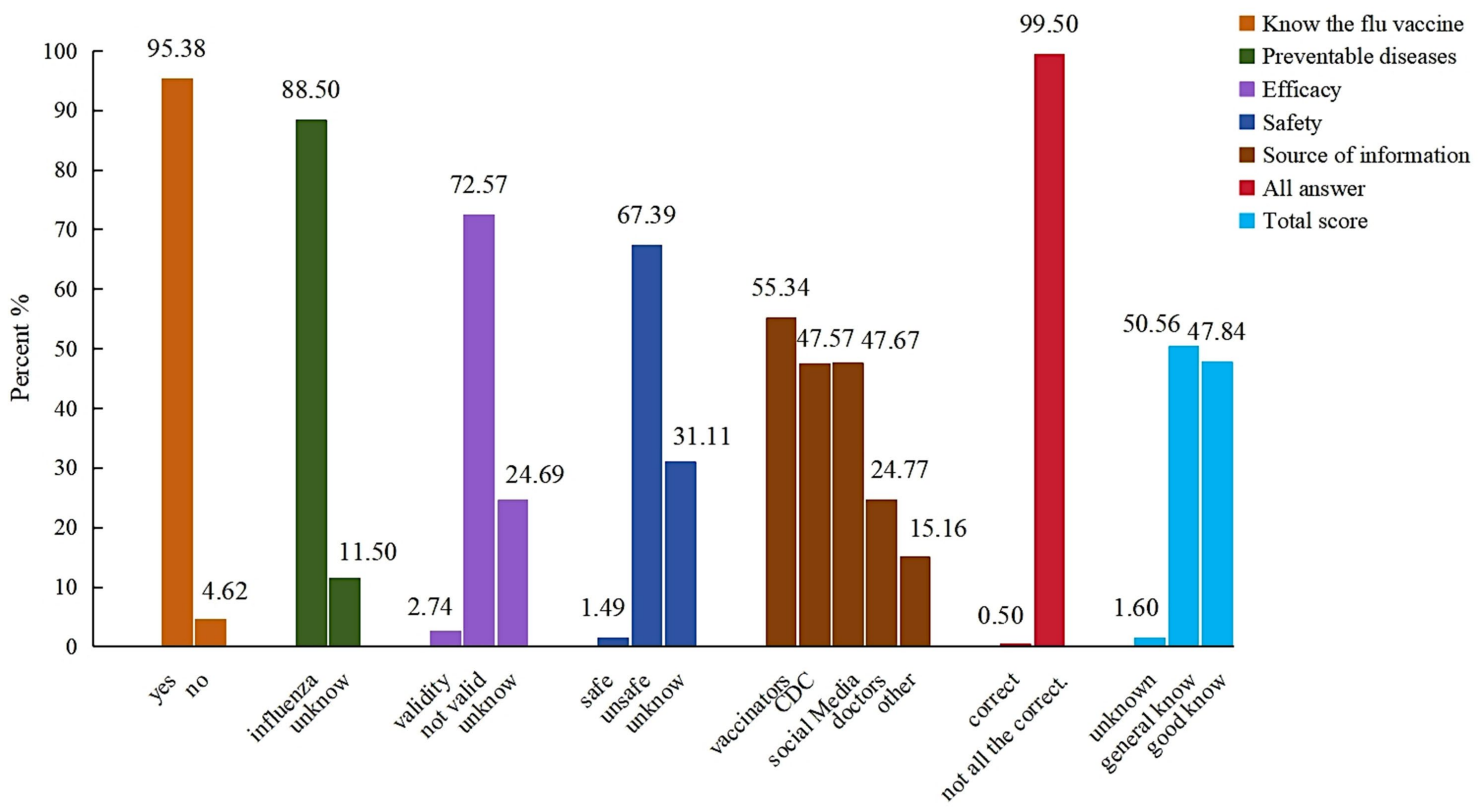
Respondents’ answers about influenza vaccine knowledge.

Figure 2 showed almost P/Gs knew the flu vaccine (95.38%), and knew that it protects against the flu (88.50%). However, only 1.49% P/Gs and 2.74% P/Gs answered “YES” about the safety and effectiveness of the influenza vaccine. When asked about the source of knowledge of flu vaccine, the main source of information about flu was vaccinators (55.34%), followed by Center for Disease Control and Prevention (CDC) (47.57%), and social media (47.67%).

Table 3 showed that almost all of P/Gs were willing to vaccinate against influenza (90.02%), main reasons for refusing the flu vaccine were concerns about the safety (47.20%), not effective (36.69%), no knowledge of vaccines (31.77%), and adverse reactions have occurred of the influenza vaccine (13.87%).

**Table 3:** Attitude toward influenza illness and influenza vaccine.

Variable	Levels	Number of respondents	Percent %
Willingness to vaccinate	Yes	7,893	90.02
	No	875	9.98
Reasons for reluctance to vaccinate (*N* = 875)	No time	59	6.74
	Self-payment required	153	17.49
	No knowledge of vaccines	278	31.77
	Worried about the safety	413	47.20
	Not effective	321	36.69
	Poor physical of children	158	18.06
	Other	138	15.77
Adverse reaction (*N* = 4,796)	No	4,131	86.13
	Fever	370	7.71
	Redness, swelling and pain at the vaccination site	340	7.09
	Hard nodules at the vaccination site	191	3.98
	Rash	83	1.73
	Other	120	2.50

### Factors associated with influenza uptake

Among preschool children respondents, 4,796 (54.70%) had received at least one dose of flu vaccine (Table 4).

**Table 4:** Vaccination rates in districts/counties in this study sample.

Variable	Levels	Vaccination *n* (%)	Average rate %
A level	Fuqing City	1,216 (52.60)	56.33
	Jinan District	461 (54.24)	
	Taijiang District	477 (58.24)	
	Yongtai County	356 (60.24)	
B level	Gulou District	465 (57.20)	55.50
	Cangshan District	520 (65.16)	
	Minhou County	932 (51.04)	
	Minqing County	369 (48.62)	
Total	Fuzhou City	4,796 (54.70)	54.70

The coverage rate of preschool children was no significant difference between strata A and B (56.33 and 55.50%, *p* > 0.05). 20–30 years old (60.70%) have higher vaccination rate for their children than 31–40 years old (51.63%) and older P/Gs (55.45%) (*p* < 0.05). The higher the education level of parents, the higher the flu vaccination rate of their children (the detailed data are in Tables 5, *p* < 0.05). The P/Gs working in state enterprises and public institutions (57.36%) had higher coverage for their children than self-employed/farmer (52.66%) and unemployed/other occupations (52.45%, *p* < 0.05). However, when we compared the household income, it showed the lower household income had higher coverage rate (*p* < 0.05). Total duration of each vaccination session < 30 min (58.72%) had a higher vaccination rate than 30–60 (52.66%) and > 60 (39.77%, *p* < 0.05) (Table 5).

**Table 5:** Univariate analysis of factors influencing influenza vaccination.

Factors	Category	Vaccination rate (%)	*β*	*p*	OR (95% CI)
Hierarchies	A	56.33			
	B	55.50	−0.02	0.69	0.94 (0.90, 1.07)
Fill the questionnaire	Mother	54.43			
	Father	54.87	0.02	0.76	1.02 (0.91, 1.14)
	Grandparents	64.49	0.42	0.02	1.52 (1.07, 2.16)
	Other	57.38	0.12	0.52	1.13 (0.79, 1.62)
Age group (years)	20–30	60.70			
	31–40	51.63	−0.37	0.00	0.69 (0.63, 0.76)
	>41	55.45	−0.22	0.01	0.81 (0.69, 0.94)
Education level	Middle school or below	52.71			
	High school	52.10	−0.02	0.73	0.98 (0.85, 1.12)
	Three-year college/university	56.01	0.13	0.04	1.14 (1.01, 1.29)
	Master and above	61.38	0.36	0.01	1.43 (1.10, 1.85)
Occupation	Enterprises and public institutions	57.36			
	Self-employed/farmer	52.66	−0.19	0.00	0.83 (0.74, 0.93)
	Unemployed/other	52.45	−0.20	0.00	0.82 (0.75, 0.90)
Average monthly household income (RMB)	<3,000	57.59			
	3,000-4,999	57.00	−0.02	0.76	0.98 (0.83, 1.14)
	5,000-9,999	53.42	−0.17	0.03	0.85 (0.73, 0.98)
	≥10,000	53.06	−0.18	0.02	0.83 (0.71, 0.98)
Rest according to legal holidays	Yes	56.60			
	Occasional overtime	52.74	−0.16	0.00	0.86 (0.78, 0.94)
	Frequent overtime	49.03	−0.30	0.00	0.74 (0.64, 0.85)
Transportation	Walking	59.72			
	Bicycle	71.15	0.51	0.02	1.66 (1.07, 2.59)
	Motorcycle/electric vehicle	54.83	−0.20	0.00	0.82 (0.71, 0.94)
	Public transport	54.15	−0.23	0.08	0.80 (0.62, 1.03)
	Self-driving	52.38	−0.30	0.00	0.74 (0.65, 0.85)
One-way inoculation time (minutes)	<30	55.16			
	30–60	52.49	−0.11	0.09	0.90 (0.79, 1.02)
	>60	43.53	−0.47	0.03	0.63 (0.41, 0.96)
Acceptable vaccine prices (RMB)	≤50	49.15			
	50–100	53.33	0.17	0.01	1.18 (1.05, 1.33)
	100–200	58.01	0.36	0.00	1.43 (1.27, 1.61)
	>200	59.50	0.42	0.00	1.52 (1.30, 1.74)
Total duration of each vaccination session	<30	58.72			
	30–60	52.66	−0.25	0.00	0.78 (0.72, 0.85)
	>60	39.77	−0.77	0.00	0.46 (0.39, 0.55)
Total score	Unknown	55.00			
	General know	57.31	0.00	0.99	1.00 (0.71, 1.41)
	Good know	53.87	−0.03	0.88	0.97 (0.69, 1.37)
Children’s characteristics					
Gender	Male	54.65			
	Female	54.76	0.00	0.92	1.00 (0.92, 1.09)
Age group	6–11 months	60.53			
	1–2 years	65.86	0.23	0.01	1.26 (1.05, 1.51)
	3–6 years	50.81	−0.40	0.00	0.67 (0.57, 0.79)
Birth order	First	58.40			
	Second	52.76	−0.23	0.00	0.80 (0.73, 0.87)
	Third or more	46.27	−0.49	0.00	0.61 (0.51, 0.73)
Cold condition	Often	55.82			
	Infrequent	54.17	−0.07	0.29	0.94 (0.83, 1.06)
	Seldom	55.89	0.00	0.97	1.00 (0.86, 1.17)
Medical insurance	No	46.53			
	Purchased	55.53	0.36	0.00	1.44 (1.24, 1.66)

Summary of children characteristics is shown in Table 5. 1–2 years old had higher vaccination rate than 6–11 months and 3–6 years old (65.86, 60.53, and 50.81%, respectively, *p* < 0.05). Furthermore, the first-born child had a higher vaccination rate than second-born and born later (58.40, 52.76, and 46.27%, respectively, *p* < 0.05). The coverage of preschool children with medical insurance was higher than that of children without (55.53 and 46.53%, respectively, *p* < 0.05).

The multivariate analysis of factors influencing flu vaccination showed that children’s birth order, medical insurance, P/G’s occupation, average monthly household income, vaccine prices, and total duration of each vaccination session were associated with flu vaccination.

Children birthed third or late [odds ratio (OR) = 0.76, 95% confidence interval (CI): 0.63, 0.92]; medical insurance [OR = 1.42, 95% CI: 1.22, 1.65]; P/Gs occupation [OR = 0.86, 95% CI: 0.76, 0.98 and OR = 0.84, 95% CI: 0.75, 0.93]; average monthly household income ≥ 10,000 [OR = 0.66, 95% CI: 0.56, 0.79]; acceptable vaccine prices > 200 [OR = 1.66, 95% CI: 1.41, 1.97]; total duration of each vaccination session > 60 min [OR = 0.49, 95% CI: 0.42, 0.58] (Table 6).

**Table 6:** Multivariate analysis of the impact of influenza vaccination.

Factors	Category	*β*	*p*	OR (95% CI)
Birth order	First			
	Second	−0.11	0.02	0.89 (0.81, 0.98)
	Third or more	−0.27	0.01	0.76 (0.63, 0.92)
Medical insurance	No			
	Purchased	0.35	0.00	1.42 (1.22, 1.65)
Age group (years)	20–30			
	31–40	−0.30	0.00	0.74 (0.67, 0.82)
	>41	−0.10	0.22	0.90 (0.77, 1.06)
Education level	Middle school or below			
	High school	−0.07	0.33	0.93 (0.81, 1.08)
	Three-year college/university	0.03	0.69	1.03 (0.89, 1.19)
	Master and above	0.29	0.05	1.33 (1.00, 1.77)
Occupation	Enterprises and public institutions			
	Self-employed/farmer	−0.15	0.02	0.86 (0.76, 0.98)
	Unemployed/other	−0.18	0.00	0.84 (0.75, 0.93)
Average monthly household income (RMB)	<3,000			
	3,000–4,999	−0.16	0.05	0.85 (0.72, 1.00)
	5,000–9,999	−0.36	0.00	0.70 (0.59, 0.82)
	≥10,000	−0.41	0.00	0.66 (0.56, 0.79)
One-way inoculation time (minutes)	<30			
	30–60	−0.04	0.54	0.96 (0.84, 1.09)
	>60	−0.27	0.23	0.76 (0.49, 1.19)
Acceptable vaccine prices (RMB)	<50			
	50–100	0.22	0.00	1.25 (1.11, 1.41)
	100–200	0.43	0.00	1.54 (1.36, 1.73)
	>200	0.51	0.00	1.66 (1.41, 1.97)
Total duration of each vaccination session	<30			
	30–60	−0.24	0.00	0.79 (0.72, 0.86)
	>60	−0.71	0.00	0.49 (0.42, 0.58)

## Discussion

Our study showed the total coverage of influenza vaccination (at least one dose) among preschool children in 2022 was 7.38%. The coverage was between 4.38–10.76% among 12 urban districts (counties). 31–40 years old P/Gs had better knowledge about influenza than younger P/Gs and older P/Gs (24.40, 21.86, and 23.52%, respectively, *p* < 0.05), but almost P/Gs were unaware of the safety and effectiveness of the influenza vaccine. This investigation showed children’s age, birth order, medical insurance, P/G’s age, occupation, average monthly household income, overtime work, transportation, vaccine prices, and total duration of each vaccination session were associated with flu vaccination.

Data obtained from the Fujian Province Immunization Information System showed the total coverage of influenza vaccination (at least one dose) among preschool children in 2022 was 7.38%, which is far below Germany (40%) (11), and England (48%) (12) who provide specialized and effective policy support for vaccination. Currently, influenza vaccination in Fuzhou operates solely on a voluntary, self-paid basis without any complementary support measures such as government subsidies or insurance coverage. This suggested deficiencies in establishing or implementing influenza vaccine policies for Fuzhou.

This investigation showed 54.70% children had received at least one dose of the influenza vaccine, which was far below the WHO’s target of 75% and domestic of 28% (13) flu vaccination coverage. However, almost P/Gs (90.02%) were willing to have their children vaccinated against the flu, which suggested they knew it’s good to get vaccinated. If all P/Gs implemented the “Willingness,” children can be protected against flu through vaccination. In this study. Higher-degree of parental had higher influenza vaccination rates among preschoolers. Larson HJ’s article indicated that parental education level is a significant predictor of vaccine uptake (14). P/Gs with high education are more likely to vaccinate their children against influenza (15). However, European study reported that lower influenza vaccination rates among highly educated individuals in Ireland, Italy, and Spain (16). This discrepancy may be attributed to cultural and cognitive differences across regions. Extensive research has established that P/Gs knowledge significantly influences childhood influenza vaccination rates (17–19). Enhancing parental understanding of influenza may consequently improve vaccination rates among children. Our research showed only 1.49% P/Gs and 2.74% P/Gs answered “YES” about the safety and effectiveness of the influenza vaccine, indicating a substantial gap in influenza- and vaccine-related knowledge. When asked about the source of knowledge of flu vaccine, the main source of information about flu was vaccinators (55.34%), followed by CDC (47.57%), and social media (47.67%). So vaccinators must provide strong advice and vaccine knowledge to P/Gs who visit vaccination clinics. Above results showed influenza coverage among preschool children was low, good knowledge of influenza and influenza vaccine were linked to improved immunization coverage. These hinted that there was still a lack of publicity about influenza.

In addition to knowledge, the convenience and feasibility of influenza vaccination are also important influencing factors. Multivariate regression analyses showed that extended vaccination time negatively impacted compliance (*β* = −0.71, *p* < 0.05). The vaccination rate was higher among the respondents who could be vaccinated within 30 min. This disparity may reflect logistical challenges P/Gs face when coordinating clinic visits with children’s schedules. Distance between outpatient clinic and home also affect the convenience of vaccination (*p* < 0.05). Furthermore, parental occupational constraints and overtime status were identified as additional barriers, Goldman (20) and Ding’s (21) articles show that it is more difficult for P/Gs to coordinate the time of vaccination for children and their work leading to low vaccination.

Furthermore, some P/Gs also showed dissatisfaction with the vaccination process, including difficulties in making vaccine appointments, long queues, and cumbersome processes. These findings suggest current influenza vaccination procedures in Fuzhou create accessibility barriers. If individuals perceive the process of getting vaccinated to be complicated or cumbersome, they may be less likely to seek it out or delay it (22, 23). To boost children influenza vaccination coverage, public health authorities should prioritize service optimization through streamlined scheduling systems, extended clinic hours, digital appointment platforms, and strengthen cooperation with the school authorities (getting vaccinated before the flu season or strengthening the promotion of flu vaccination in schools).

Our results showed there was a significant inverse correlation between household income and influenza vaccination rates (*β* = −0.41, *p* < 0.05), which coincides with previous findings that vaccination rates among children tend to be higher in families with lower economic status (24). This phenomenon may be attributed to the greater perceived cost-effectiveness of immunization for economically disadvantaged families, where disease prevention represents a more substantial economic benefit. Health insurance coverage among preschool children is significantly associated with increased influenza vaccination uptake (*p* < 0.05). In addition, Fuzhou’s self-funded vaccination policy makes socially and economically disadvantaged groups have the burden of vaccination. The price of vaccines is an important deterrent to vaccination behavior. Comparative analyses of vaccination policies reveal that regions implementing free vaccination programs, such as Beijing and Hong Kong, achieve significantly higher coverage rates. Comparative analyses of vaccination policies reveal that regions such as Beijing (25) and Hong Kong (26), which implement free vaccination policy achieved significantly higher coverage rates than Fuzhou. Empirical evidence from national policy evaluations confirms that free vaccination policies yield the highest coverage rates, followed by medical insurance reimbursement systems (27, 28). These findings underscore the critical role of policy interventions and financial support mechanisms in enhancing vaccination uptake. In order to comprehensively increase influenza vaccine coverage among school-aged children in Fuzhou, we recommend implementing comprehensive strategies including: (1) establishment of free or subsidized vaccination programs, (2) integration with existing healthcare insurance systems, and (3) development of targeted initiatives for vulnerable populations.

Our study had a few limitations. First, the data on vaccination were self-reported, and not verified by immunization certificate, document, or serological testing. Second, the survey is a self-administered questionnaire by the respondents, we relied on respondents to fill out the questionnaire themselves to complete the survey, which may have limited a small percentage of P/Gs from responding.

In conclusion, our study offers valuable insights into the determinants influencing Chinese P/Gs’ willingness to vaccinate influenza vaccine for their children. The findings demonstrate that as factors such vaccine knowledge, medical insurance, occupation, household income, overtime work, transportation, vaccine prices, and total duration of each vaccination session play a pivotal role in shaping P/Gs’ intentions to influenza vaccine. Public health campaigns and educational initiatives should emphasize the benefits associated with the influenza vaccine. First, public health authorities should establish sustainable financing mechanisms and incentive programs to ensure vaccine accessibility and affordability, health and education departments can do a good job of health education on influenza and vaccination in advance, improve the corresponding cognitive level of the target population, convert vaccination intentions into actual uptake. These integrated measures, when implemented prior to influenza seasons, could significantly improve vaccination rates among preschool children, thereby establishing herd immunity and reducing influenza-related morbidity.

## Data Availability

The original contributions presented in the study are included in the article/supplementary material, further inquiries can be directed to the corresponding author.
